# Supplementary material for: Peripherally Inserted Central Venous Catheter (PICC) Related Bloodstream Infection in Cancer Patients Treated with Chemotherapy Compared with Noncancer Patients: A Propensity-Score-Matched Analysis
Source: Cancers (Basel). 2023 Jun 20;15(12):3253. doi: 10.3390/cancers15123253 (PMC10296793; doi:10.3390/cancers15123253)
Supplement: Supplementary file 1 [file cancers-15-03253-s001.zip › cancers-2403322-supplementary.pdf]

cancers

Supplementary materials

Peripherally Inserted Central Venous Catheter (PICC) Related Bloodstream Infection in Cancer Patients Treated with Chemotherapy Compared with Noncancer Patients: A Propensity-Score-Matched Analysis

Romaric Larcher, Koko Barrigah-Benissan, Jerome Ory, Claire Simon, Jean-Paul Beregi, Jean-Philippe Lavigne and Albert Sotto

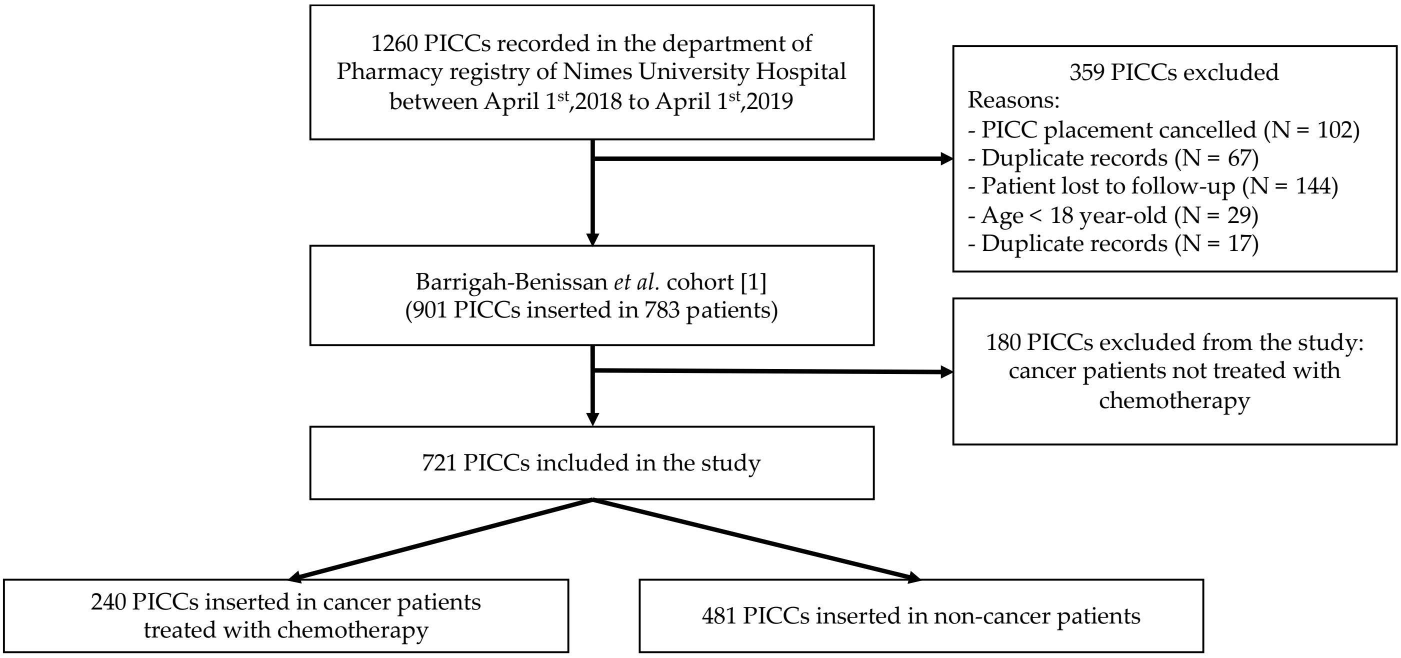

Figure S1. Flow chart of the study population and peripherally inserted central catheters (PICC).

Table S1. Bacterial species involved in peripherally inserted central catheter (PICC) colonization and non-bacteremia PICC-related infection.

**Bacterial species**	**Overall [1]**	**Cancer patients [1]**	**Non-cancer patients [1]**
PICC colonization			
Gram-positive bacteria	32 (97%)	13 (87%)	18 (100%)
Staphylococcus epidermidis	21 (64%)	10 (67%)	11 (61%)
Staphylococcus haemolyticus	5 (15%)	1 (6.7%)	4 (22%)
Staphylococcus capitis	2 (6.1%)	1 (6.7%)	1 (5.6%)
Staphylococcus hominis	2 (6.1%)	0 (0%)	2 (11%)
Staphylococcus warnerii	1 (3%)	0 (0%)	1 (5.6%)
Corynebacterium sp.	1 (3%)	1 (6.7%)	0 (0%)
Gram-negative bacteria	3 (9.1%)	2 (13%)	1 (5.6%)
Escherichia coli	2 (6.1%)	2 (13%)	0 (0%)
Morganella morganii	1 (3%)	0 (0%)	1 (5.6%)
NB-PICCRI			
Gram-negative bacteria	4 (0.6%)	2 (0.8%)	2 (0.4%)
Pseudomonas aeruginosa	2 (0.3%)	1 (0.4%)	1 (0.2%)
Pseudomonas putida	1 (0.1%)	0 (0%)	1 (0.2%)
Serratia marcescens	1 (0.1%)	1 (0.4%)	0 (0%)
Gram-positive bacteria	3 (0.4%)	2 (0.8%)	1 (0.2%)
Staphylococcus aureus	3 (0.4%)	2 (0.8%)	1 (0.2%)
Fungi	4 (0.6%)	1 (0.4%)	3 (0.6%)
Candida albicans	3 (0.4%)	0 (0%)	3 (0.6%)
Candida parapsilosis	1 (0.1%)	1 (0.4%)	0 (0%)

[1] N (%); [2] NB-PICCRI: non-bacteremia PICC related infection
